# Supplementary figures and images for: A comparison of neuronal population dynamics measured with calcium imaging and electrophysiology
Source: PLoS Comput Biol. 2020 Sep 15;16(9):e1008198. doi: 10.1371/journal.pcbi.1008198 (PMC7518847; doi:10.1371/journal.pcbi.1008198)

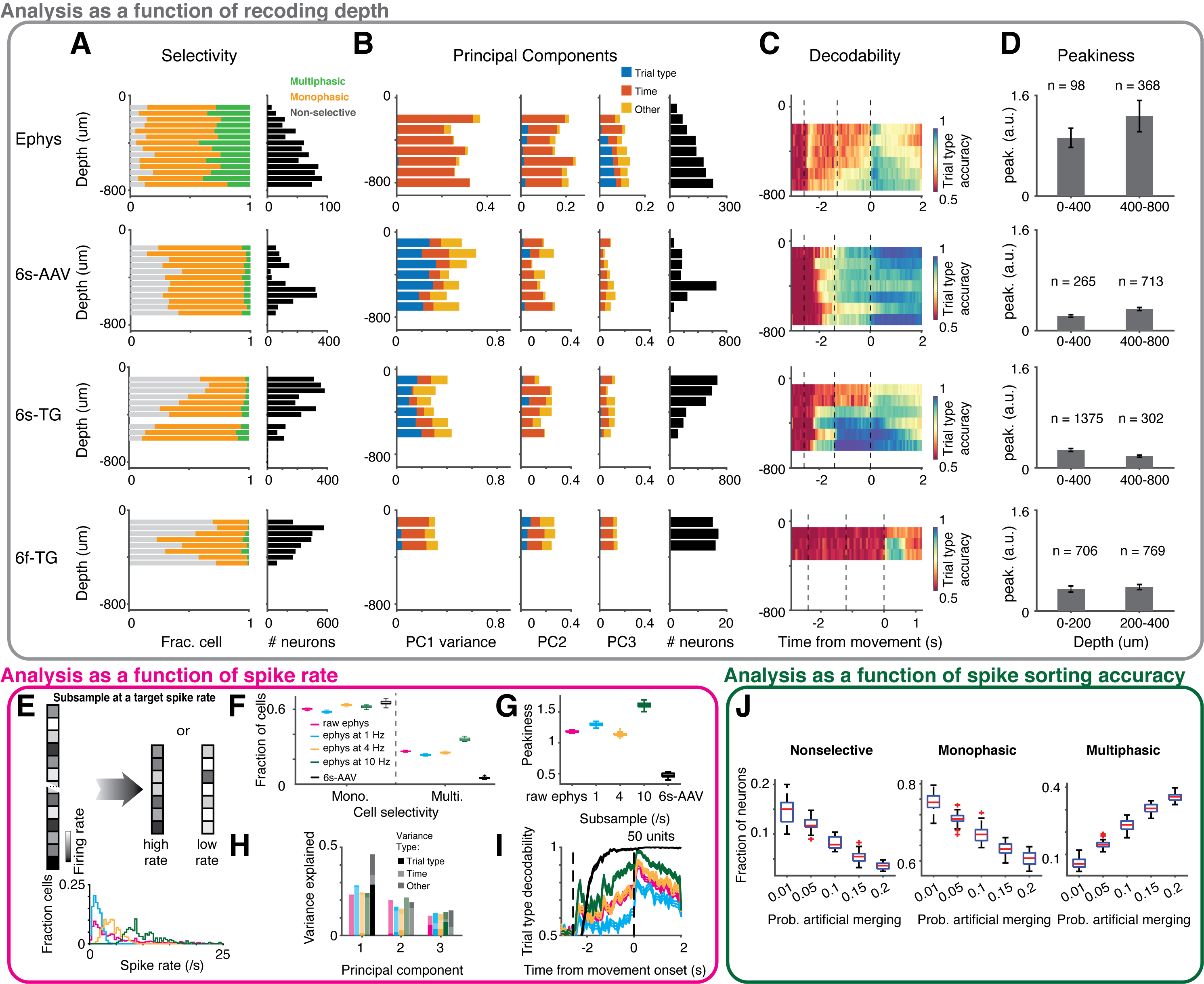

Supplement: S1 Fig — A-D. Analysis as a function of recording depth. A. Single neuron selectivity-type analyses. Left: horizontal bar plots show breakdown of the population into selectivity types (gray: non-selective neurons, orange: monophasic-selective neurons, green, multiphasic-selective neuron. Right: horizontal bar plot shows number of neurons at each depth. The ratio of monophasic- to multiphasic selective neuron was similar across depths (χ2-test to depths with n > 50 cells, ephys: p = .19; 6s-AAV: p = .73; 6s-TG: p = .97; 6f-TG: p = .43). For the same depth, ephys has more selective neurons and more multiphasic selective neurons than imaging (χ2-test, p < .001 for all). B. Percentage of variance of neural activity explained by each principal component (Fig 5). Left: length of horizontal bar shows fraction of variance in each principal component. Colors show breakdown into different types of variance (blue: trial-type, red: time, orange: other). Right: horizontal bar shows number of neurons in each depth. For the same depth, the 1st PC show more temporal dynamics content in ephys and 6f-TG (χ2-test, p < .001 for all), while that show more trial-type content in 6s-AAV and 6s-TG (χ2-test, p < .001 for all). C. Decodability of trial type (Fig 6). The number of cells at each depth is identical to that in PCA analyses. The decodability differs across depths, where the neurons in superficial layers show weak decodability of trial type in sample-delay epoch (multivariate ANOVA test on time-series to depths with n > 50 cells in ephys, 6s-AAV and 6s-TG; that to depth with n > 10 cells at ROC > 0.7 in 6f-TG; p < .001, 1000 bootstrap). For the same depth, the average decodability of trial type is higher in late delay to early response in imaging than that in ephys (rank sum test, p < .001 for all, 1000 bootstrap). D. Peakiness (Fig 7). The peakiness differs across depths (rank sum test, p < .001, 1000 bootstrap). For the same depth, peakiness is higher in ephys than imaging (rank sum test, [file pcbi.1008198.s001.tif]

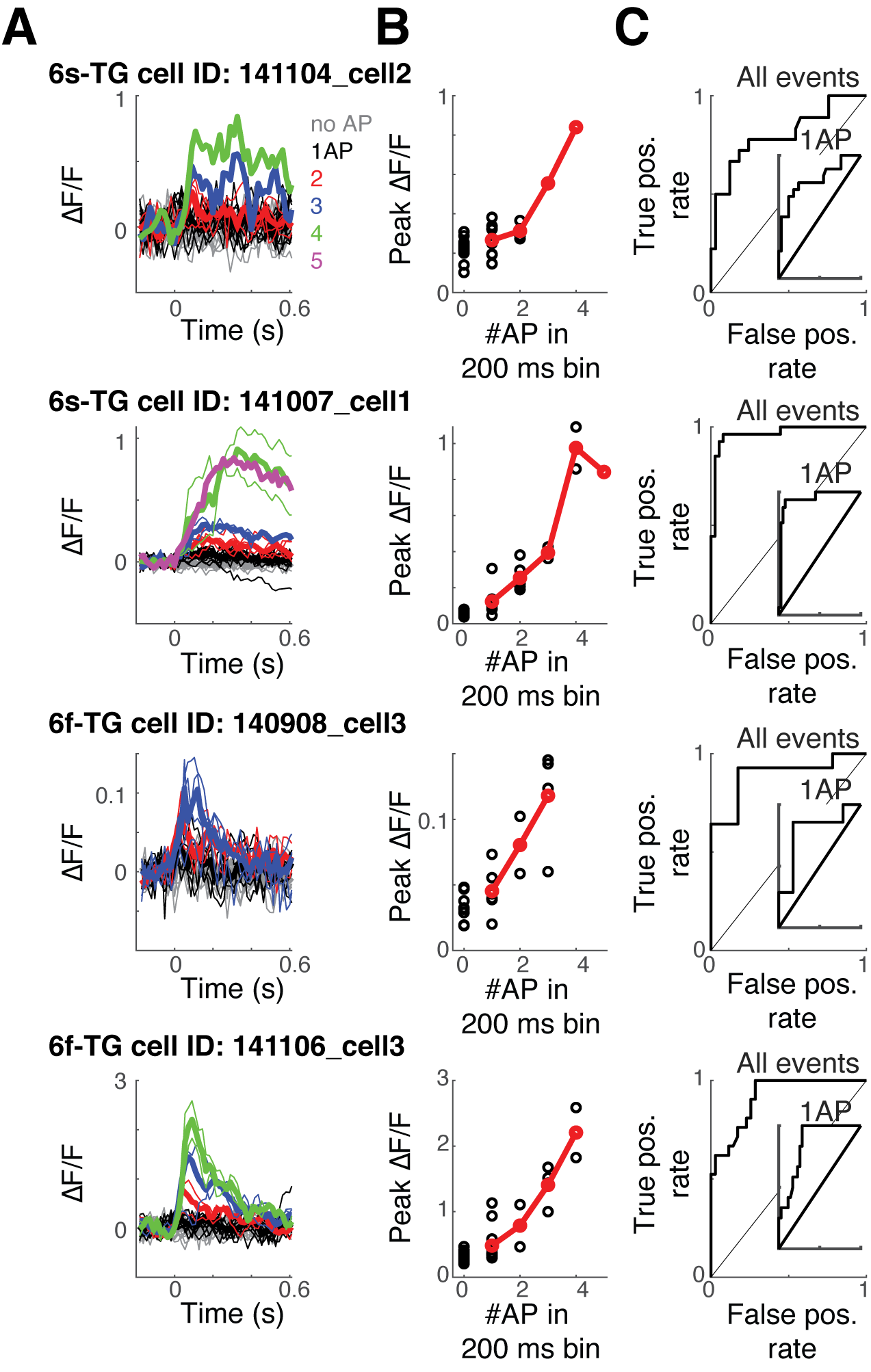

Supplement: S2 Fig — A. Traces of fluorescence dynamics following different numbers of action potentials (APs) for example neurons (same plots as Fig 3C for additional examples). Gray, no AP; black, a single AP; red, 2 APs; blue, 3APs; green, 4APs; magenta, 5APs. Thin lines, single trials; thick lines, average. B. Peak fluorescence change as a function of the number of spikes (same plots as Fig 3D for additional examples). Black, single trials; red, trial average. C. ROC curve of all spike events. Inner panel, ROC curve for single AP events (same plots as Fig 3E for additional examples). (TIF) [file pcbi.1008198.s002.tif]

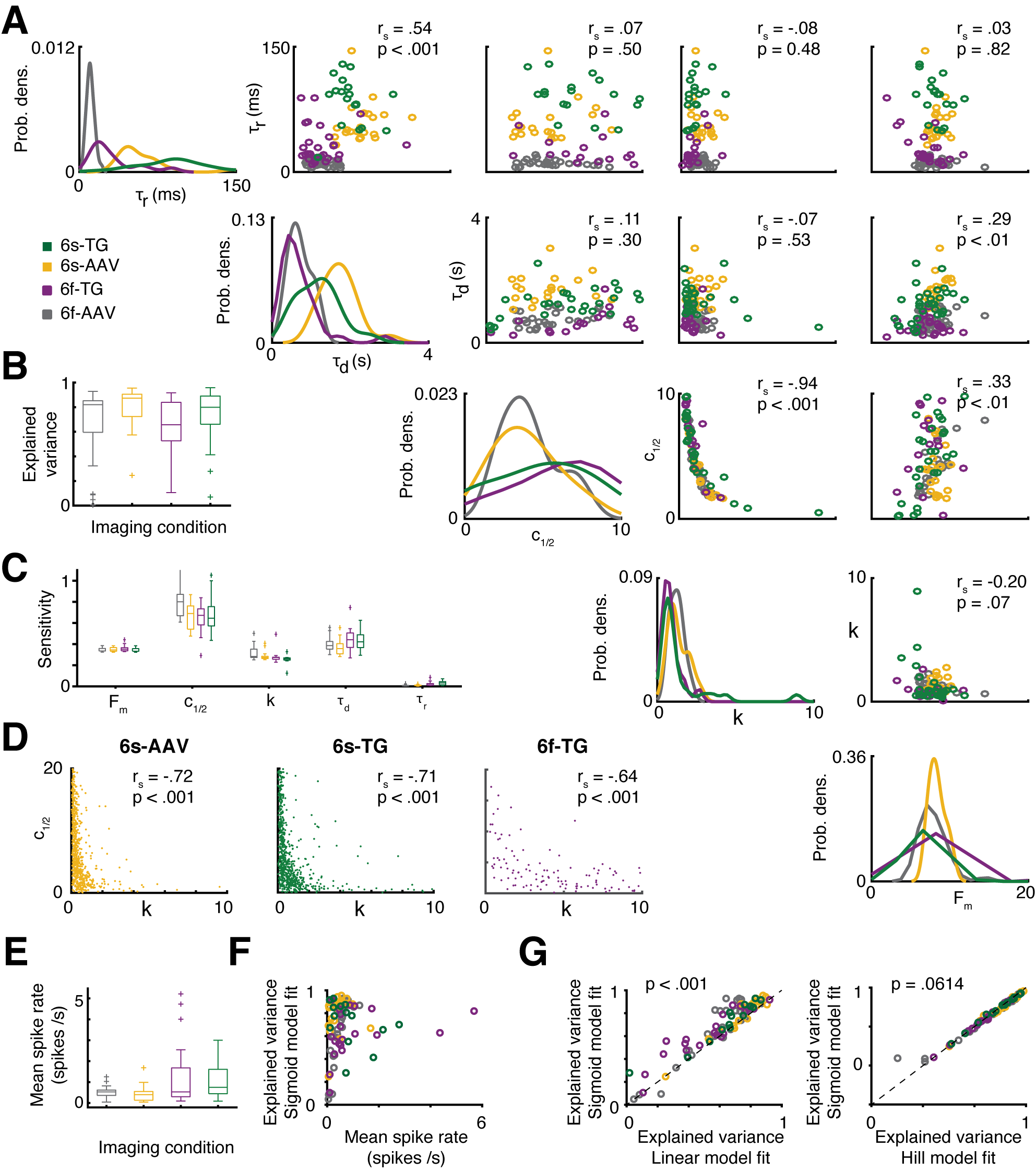

Supplement: S3 Fig — A. Pairwise correlation plots for each of the spike-to-fluorescence parameters. Panels along the diagonal describe the distribution of each parameter (these are identical to Fig 4B but reproduced to facilitate comparisons). Off-diagonal panels depict the correlation between two parameters. Spearman’s rank correlation of parameters across cells (regardless of recording method) and associated p-value are provided in each off-diagonal panel. Each circle corresponds to a response set. Data from the different indicator conditions is overlaid and marked by color. (gray: 6f-AAV, 11 neurons, 37 response sets; yellow: 6s-AAV, 9 neurons, 21 response sets; purple: 6f-TG, 18 cells, 32 response sets; green: 6s-TG, 22 neurons, 33 recording periods). B. Boxplots of explained variance of S2F on validation data for simultaneously recorded neurons (color follows the same convention as in A). C. Boxplot of distribution of parameter sensitivity values. D. Pairwise correlation of re-estimation of k and c1/2 using ALM imaging dynamics (Materials and methods). The re-estimated parameter values are shown as a scatter plot. Each dot corresponds to a neuron (n = 720 for 6s-AAV and 6s-TG; n = 225 for 6f-TG in matched depths). The distribution of the re-estimated parameter values strongly overlapped with those obtained in simultaneous imaging-ephys recordings. c1/2 and k had a strong inverse correlation as in the simultaneously recorded data (rs < -.64, p < .001). E. Boxplots of firing rates of neurons in each recording sessions (6f-AAV, gray, 0.51 ± 0.25 Hz, mean ± std., range 0.05–1.25 Hz; 6s-AAV, yellow, 0.43 ± 0.38 Hz, range 0.05–1.68 Hz; 6f-TG, purple, 1.25 ± 1.48 Hz, range 0.09–5.22 Hz; 6s-TG, green, 1.08 ± 0.85 Hz, range 0.09–3.00 Hz). F. Scatter of simultaneous ephys-imaging data model fit and the dynamical range of the data (expressed as mean spike rate). G. Scatter of simultaneous ephys-imaging data model fit quality between different S2F models (Materials and methods). Left: compari [file pcbi.1008198.s003.tif]

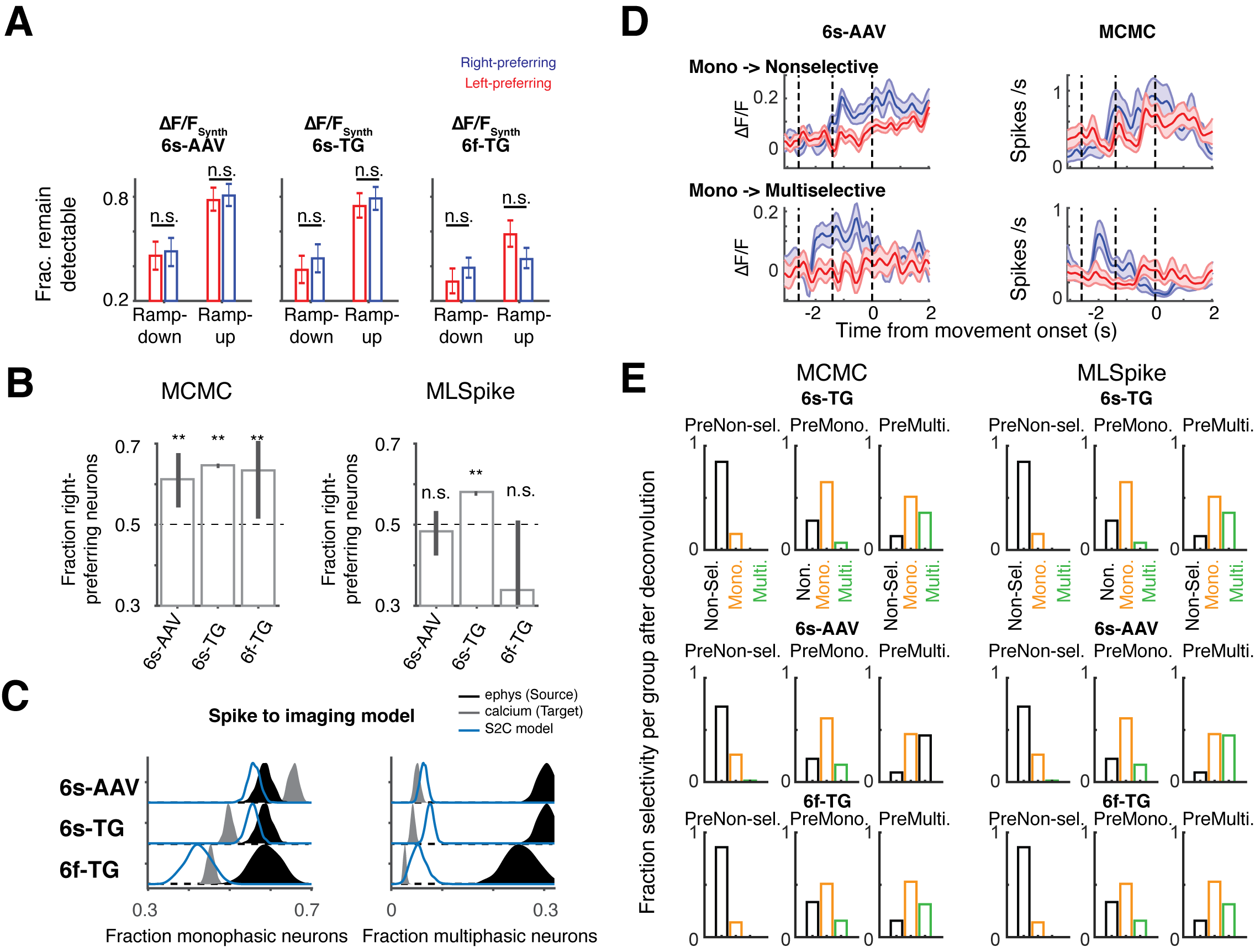

Supplement: S4 Fig — A. Fraction of cells that remain selective in synthetic imaging plotted separately for ramp-down and ramp-up cells (left: 6s-AAV synthetic, middle: 6s-TG synthetic, right: 6f-TG synthetic), which is further broken down into right- (blue) and left-preferring (red) trials. B. Fraction of right-preferring neurons in imaging after spike inference models. Left: the same analyses as that in Fig 2I, but performed on inferred spiking data obtained via the MCMC framework; right: the same analyses as that in Fig 2I, but performed on inferred spiking data obtained via the MLSpike framework. C. Estimation of the fraction of monophasic and multiphasic neurons that would be discovered by an imaging experiment through use of the S2F forward model. Plots show the estimates for monophasic (left) and multiphasic (right) neurons. The proportion of the source data, ephys, is in black. The experimentally measured proportions in imaging are in gray. Blue color shows the distribution of selectivity type proportion for different repetitions of each algorithm on subsamples of the dataset for synthetic imaging using 6s-AAV (top), 6s-TG (middle) and 6f-TG (bottom) parameters. D. Example neurons that change their selectivity after F2S models. Top, a mono-phasic neuron becomes nonselective after F2S model; bottom, a mono-selective neuron becomes multi-phasic after F2S model. E. Fraction of selectivity change per selective group after F2S models. Left, MCMC F2S model; right, MLSpike F2S model. Top, 6s-TG imaging; middle, 6s-AAV; bottom, 6f-TG. First column, non-selective neurons before F2S model; second column, mono-phasic; third column, multi-phasic. First bar, non-selective neurons after F2S model; second bar, mono-phasic; third bar, multi-phasic. (TIF) [file pcbi.1008198.s004.tif]

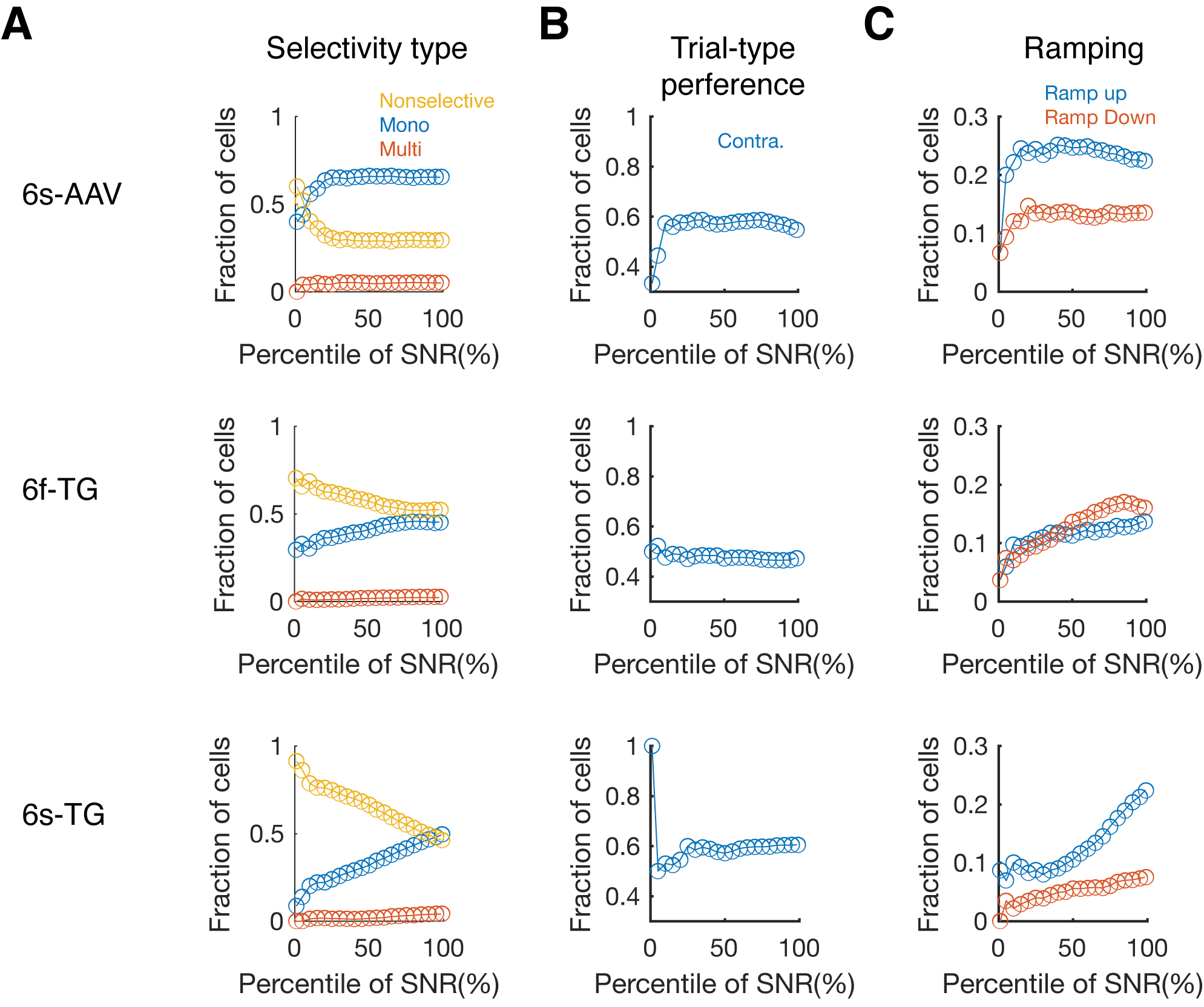

Supplement: S5 Fig — We estimated SNR using the procedure in the widely used CaImAn package, in which the noise level is estimated as the exponential of the mean of the logarithm of power spectral density. We then generated datasets including only a subset of neurons by moving the threshold up from its zeroth percentile to its 100th percentile. A. non-selective (yellow), mono-selective (blue) and multi-selective neurons (red). Top, 6s-AAV imaging; middle, 6f-TG; right, 6s-TG. B. Contra-selective (blue). The remaining was ipsi-selective neurons. C. Ramp-up (blue) and ramp-down (red). The remaining was other neurons. (TIF) [file pcbi.1008198.s005.tif]

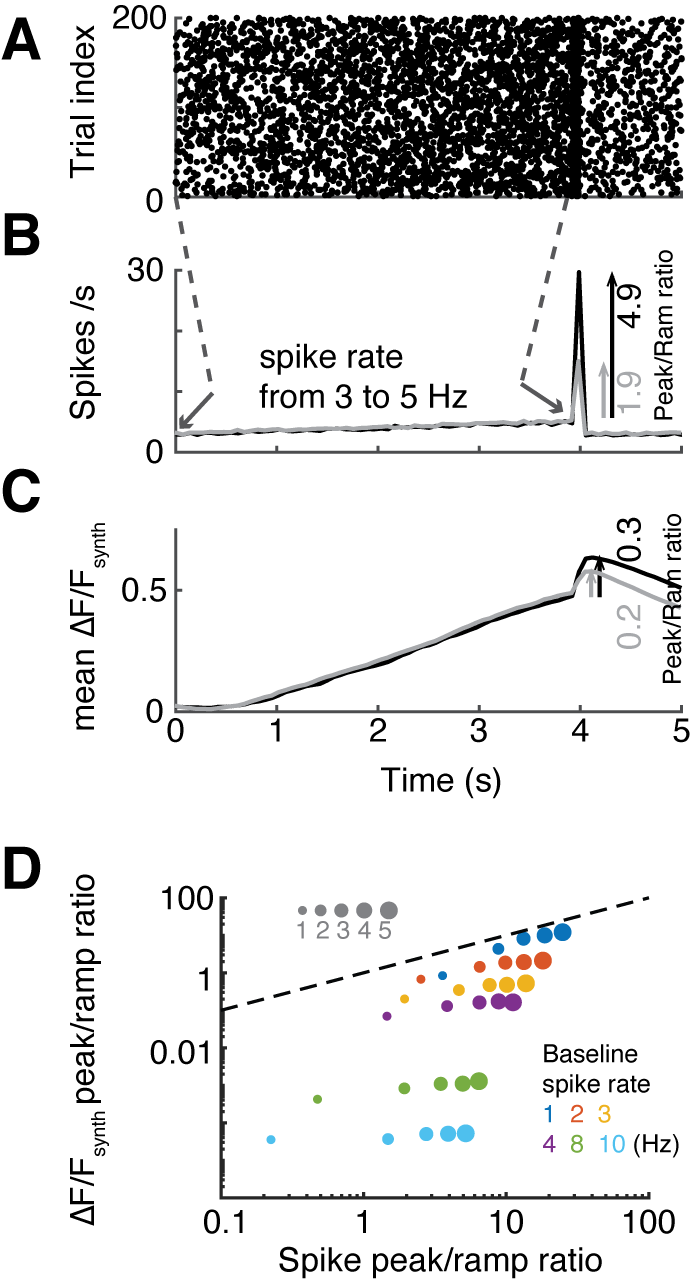

Supplement: S6 Fig — An important assumption of F2S models is that baseline fluorescence reflects zero spikes. This assumption is rarely met. For example, in our study, ALM neurons fire at about 6 Hz in the pre-sample period. This background firing rate, which can vary across time and from neuron to neuron, can distort measures of neural dynamics based on imaging. We explored this effect using computer simulations. The firing rate of a simulated neuron (baseline at 3 Hz) was gradually increased by 2 Hz over four seconds followed by a brief phasic response (1 to 5 spikes were evoked in 70 ms; Fig S6A). We computed the peak over ramp ratio (i.e. ratio of the maximum firing rate during phasic firing to the maximum firing rate before phasic firing) as the measure of the detectability of the phasic activity from tonic activity. We found that the small change of the tonic activity became prominent while detectability of phasic activity was reduced by a factor of >10 in calcium imaging (Fig S6BC). This stems from the integration in calcium dynamics. Although the ramping activity was weak, it was integrated over seconds; although the phasic activity was strong, it was only integrated over 100 ms. The degree to which the detectability was reduced in imaging (comparing to ephys) increased with the level of baseline spike rate (Fig S6D). Therefore, baseline subtraction can be problematic for inference when the underlying baseline spike rate is unknown. A. Simulation (200 trials) of a single neuron, whose firing rate slowly increased from 3 Hz to 5 Hz over ~4 seconds and was then followed by a transient increase (phasic firing) to 15Hz or 30 Hz in 70 ms, and then reset to 3 Hz (baseline). Black dots, spike events; gray dash line, onset time of transient increased spike events. B. Spike rate. Black line, phasic firing at 30 Hz; gray, phasic firing at 15 Hz. C. Mean ΔF/Fsynth from S2F model. The change of fluorescence came more from the small change of the baseline firing, and little from the strong [file pcbi.1008198.s006.tif]

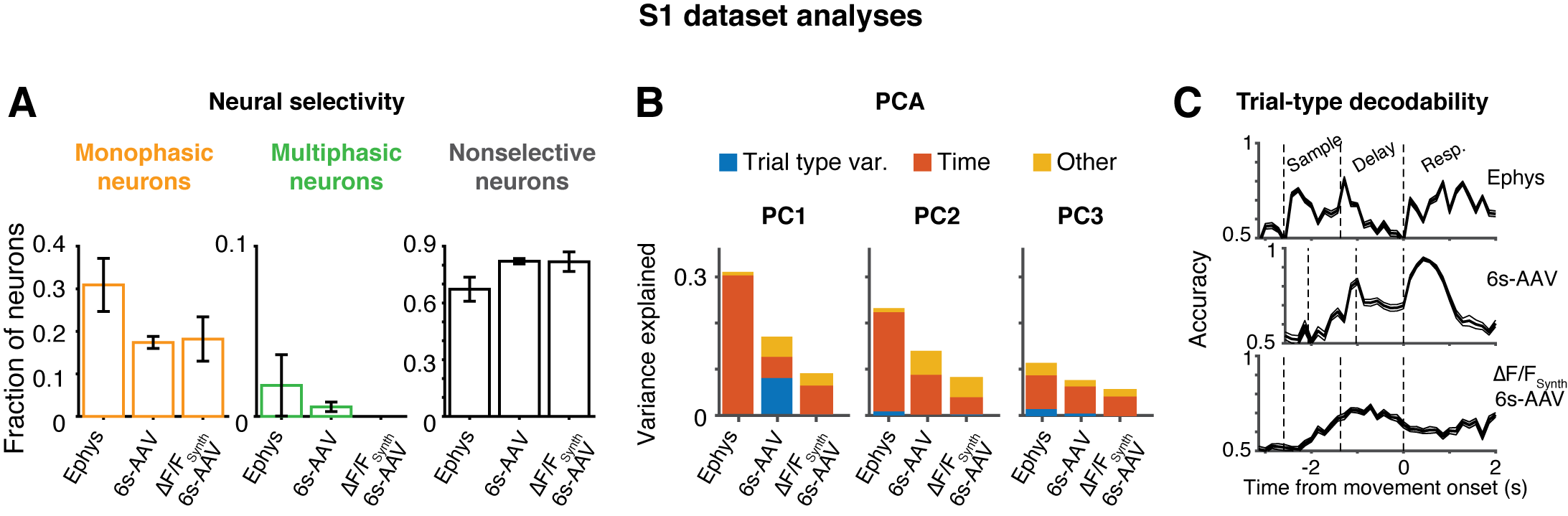

Supplement: S7 Fig — Differences between ephys and imaging are likely to depend not only on the analysis and indicator, but also on the underlying dynamics which change from one brain area to the other. We analyzed a second group of matched population recordings, obtained from primary somatosensory area (S1) rather than ALM. We find that differences in some analyses were no longer present, but others remained. We find that the fraction of multiphasic neurons in S1 was far smaller than that in ALM (n = 1/55, ephys; n = 4/719, 6s-AAV; p < .001, χ2 test) and there was no significant difference between the fraction of multiphasic neurons observed in ephys and imaging (p = .801, χ2 test). Our forward model correctly predicted this lack of change (p = .674, χ2 test between imaging data and synthetic imaging data). Similarly to ALM data, trial type variance dominated the first principal component in imaging but not in ephys and population decoding was substantially delayed in imaging relative to ephys. A. Single neuron selectivity type. Bar plots show fraction of neurons found in each of the three selectivity types (left: monophasic, middle: multiphasic, right: nonselective) for the different recording methods (left: ephys, middle: 6s-AAV, right: 6s-AAV synthetic). B. principal component variance content. Bar plots show fraction of variance contained in the first three principal components (from left to right: PC1, PC2, PC3). Each bar is broken into the contribution from trial-type variance (blue), time variance (red) and other (yellow). C. Population trial-type decodability. Plot shows mean decodability over time for ephys: top, 6s-AAV: middle and synthetic 6s-AAV: bottom. Dashed lines designate different trial periods (sample, delay response). Note that the experiments with 6s-AAV had a slightly shorter delay period, hence the difference in location of dashed lines. Since 6s-AAV synthetic is derived from ephys it has the same trial structure as ephys. (TIF) [file pcbi.1008198.s007.tif]

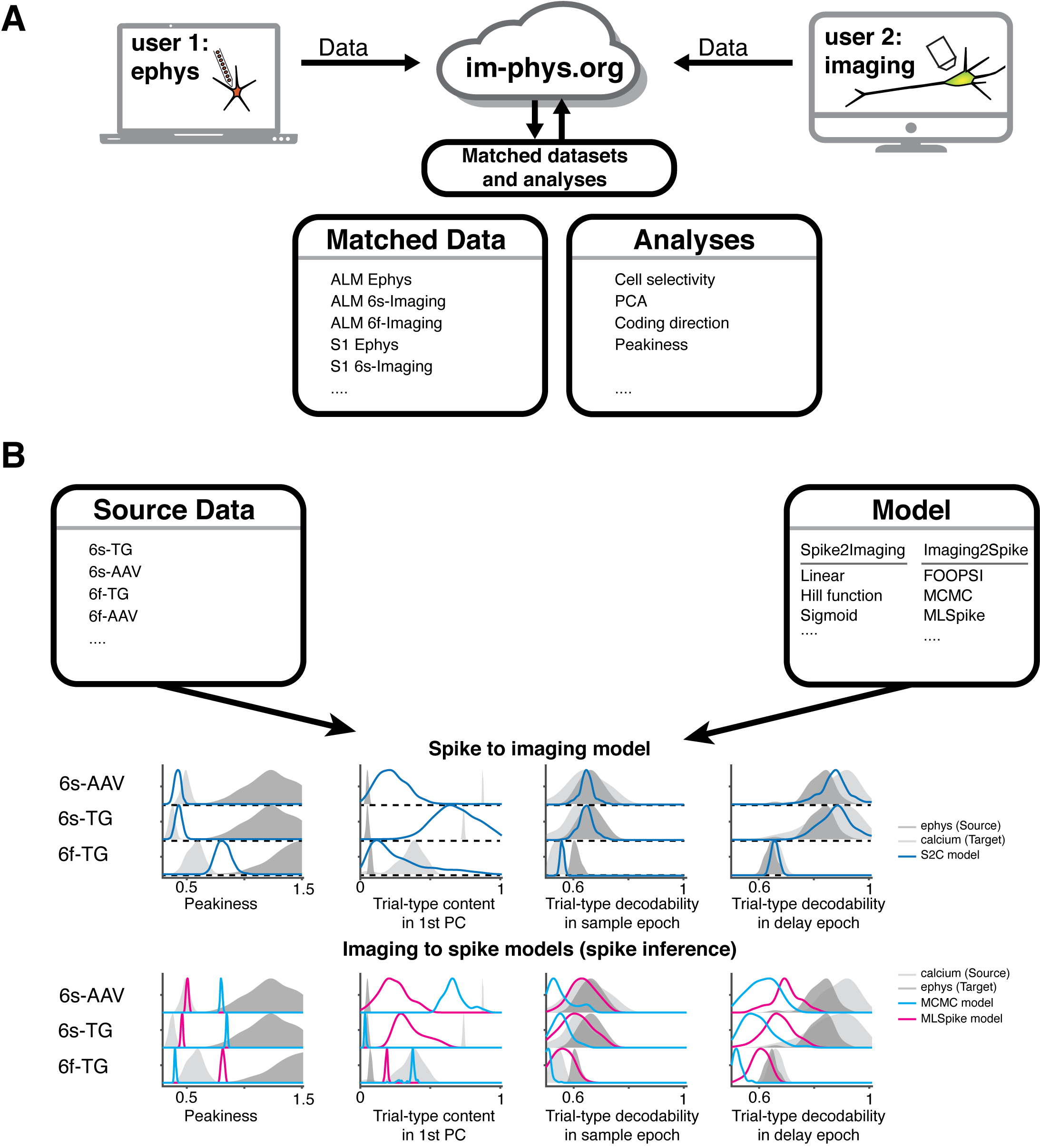

Supplement: S8 Fig — A. Top, schematic of our community resource that can allow datasets acquired by different labs to be found in one location and matched in analyses. Bottom, schematic of combining different analyses with different datasets on im-phys.org. B. Schematic of using im-phys.org to predict values (metric distributions) expected for different population analyses from datasets acquired by different techniques through use of a variety of forward and inverse models. (TIF) [file pcbi.1008198.s008.tif]
