# Supplementary material for: A comparison of neuronal population dynamics measured with calcium imaging and electrophysiology
Source: PLoS Comput Biol. 2020 Sep 15;16(9):e1008198. doi: 10.1371/journal.pcbi.1008198 (PMC7518847; doi:10.1371/journal.pcbi.1008198)
Supplement: S1 Table — List of datasets. Includes type of dataset, number of neurons, link to dataset, figures in manuscript and citation for data. (DOCX) [file pcbi.1008198.s009.docx]

**Summary of large-scale electrophysiological and imaging recordings**

| **Datasets** | **Ephys/GCaMP** | **# cells** | **DOI/external link** | **Figure** | **Citation** |
| --- | --- | --- | --- | --- | --- |
| Li-ALM#1 | Ephys | 720 | <http://crcns.org/data-sets/motor-cortex/alm-1> | 2, 4-7, S1, S4-S6 | Li & Chen et al., Nature, 2015 |
| Inagaki-ALM#1 | Ephys (intra-) | 35 | <https://doi.org/10.6084/m9.figshare.12786296.v1> | 2G | Guo & Inagaki et al., Nature, 2017 |
| Chen-ALM#1 | GCaMP6s-AAV | 1493 | <http://crcns.org/data-sets/motor-cortex/alm-2> | 2, 4-7, S1, S4-S6 | Li & Chen et al., Nature, 2015 |
| Chen-ALM#2 | GCaMP6s-TG | 2293 | <https://doi.org/10.6084/m9.figshare.12786296.v1> | 2, 4-7, S1, S4-S6 | Chen et al., Neuron, 2017 |
| Daie-ALM#1 | GCaMP6f-TG | 2672 | <https://doi.org/10.6084/m9.figshare.12786296.v1> | 2, 4-7, S1, S4-S6 | Wei et al. (this manuscript) |
| Li-S1#1 | Ephys | 55 | <https://doi.org/10.6084/m9.figshare.12786296.v1> | S7 | Guo & Li et al., Neuron, 2014 |
| Peron-S1#1 | GCaMP6s-AAV | 719 | <http://crcns.org/data-sets/ssc/ssc-2> | S7 | Peron et al., Neuron, 2015 |
| Lin-V1#1 | Simultaneous Ephys-GCaMP6-TG | 40 | <https://doi.org/10.6084/m9.figshare.12786296.v1> | 3, S2, S3 | Wei et al. (this manuscript) |
| Chen-V1#1 | Simultaneous Ephys-GCaMP6-AAV | 20 | <http://crcns.org/data-sets/methods/cai-1> | S3 | Chen et al., Nature, 2013 |
