# Supplementary material for: A comparison of neuronal population dynamics measured with calcium imaging and electrophysiology
Source: PLoS Comput Biol. 2020 Sep 15;16(9):e1008198. doi: 10.1371/journal.pcbi.1008198 (PMC7518847; doi:10.1371/journal.pcbi.1008198)
Supplement: S2 Table — List of single neurons recorded simultaneously by ephys and imaging. Includes duration of recording, spike rate properties and inferred decay time constant of calcium imaging. (DOCX) [file pcbi.1008198.s010.docx]

**Summary of simultaneous ephys-imaging recordings in GCaMP6s-TG mice**

| **Cell ID** | **Recording duration (s)** | **Mean spike rate (Hz)** | **Mean spike rate during stimulus (4 s) (Hz)** | **Max spike rate in 100-ms bins (Hz)** | **Inferred decay time const. (sec)** |
| --- | --- | --- | --- | --- | --- |
| 141001_cell1 | 292 | 0.26 | 4 | 40 | 2.55 |
| 141001_cell2 | 1067 | 0.92 | 9.5 | 100 | 1.13 |
| 141002_cell1 | 312 | 2.2 | 16.25 | 90 | 1.58 |
| 141002_cell2 | 156 | 0.75 | 3 | 20 | 1.37 |
| 141002_cell3 | 156 | 2.23 | 7.25 | 70 | 1.17 |
| 141002_cell4 | 412 | 1.76 | 9 | 60 | 2.02 |
| 141006_cell1 | 186 | 2.27 | 7.5 | 60 | 0.53 |
| 141006_cell2 | 132 | 0.4 | 4.75 | 90 | 0.40 |
| 141007_cell1 | 526 | 0.76 | 6.5 | 50 | 1.33 |
| 141007_cell3 | 1315 | 0.15 | 3.25 | 40 | 1.11 |
| 141007_cell4 | 443 | 3.39 | 10 | 40 | 0.55 |
| 141010_cell1 | 526 | 0.34 | 5 | 70 | 1.73 |
| 141010_cell2 | 165 | 0.29 | 2 | 50 | 1.56 |
| 141010_cell3 | 526 | 0.33 | 1.5 | 40 | 1.45 |
| 141010_cell5 | 611 | 3.19 | 13 | 60 | 0.79 |
| 141028_cell1 | 160 | 1.68 | 12.5 | 70 | 0.93 |
| 141029_cell1 | 363 | 2.67 | 6.25 | 50 | 0.56 |
| 141029_cell2 | 100 | 0.84 | 4.5 | 70 | 0.62 |
| 141029_cell4 | 526 | 1.89 | 8.5 | 60 | 1.90 |
| 141104_cell1 | 263 | 0.47 | 5.25 | 50 | 1.23 |
| 141104_cell2 | 766 | 2.31 | 14.25 | 70 | 0.90 |
| 141104_cell3 | 761 | 0.96 | 7.25 | 70 | 0.78 |
| N=22 | 444±316 | 1.37±1.02 | 7.32±4.01 | 60±19 | 1.19±0.55 |

**Summary of simultaneous ephys-imaging recordings in GCaMP6f-TG mice**

| **Cell ID** | **Recording duration (s)** | **Mean spike rate (Hz)** | **Mean spike rate during stimulus (4 s) (Hz)** | **Max spike rate 100-ms bins (Hz)** | **Decay time (sec)** |
| --- | --- | --- | --- | --- | --- |
| 140822_cell1 | 240 | 0.29 | 3 | 100 | 0.39 |
| 140822_cell2 | 240 | 4.24 | 15 | 70 | 0.89 |
| 140826_cell1 | 100 | 0.7 | 3.25 | 40 | 0.23 |
| 140828_cell1 | 248 | 0.45 | 11.5 | 80 | 0.35 |
| 140904_cell2 | 744 | 0.59 | 8.5 | 220 | 0.41 |
| 140904_cell3 | 50 | 2.46 | 5 | 60 | 0.31 |
| 140908_cell1 | 744 | 2.19 | 11.75 | 70 | 0.81 |
| 140908_cell3 | 120 | 0.9 | 7.75 | 50 | 0.53 |
| 140911_cell1 | 403 | 1.11 | 5.5 | 40 | 0.69 |
| 141110_cell1 | 526 | 0.4 | 3 | 60 | 0.41 |
| 141110_cell2 | 789 | 0.95 | 8.25 | 50 | 0.85 |
| 141110_cell3 | 263 | 1.46 | 11.75 | 110 | 0.70 |
| 141113_cell1 | 263 | 1.89 | 12 | 70 | 0.91 |
| 141113_cell2 | 526 | 0.7 | 6 | 70 | 1.17 |
| 141113_cell3 | 263 | 1.76 | 8.5 | 50 | 0.39 |
| 141113_cell4 | 2630 | 0.54 | 8.5 | 70 | 1.04 |
| 141114_cell1 | 1152 | 4.35 | 18.75 | 80 | 0.67 |
| 141114_cell2 | 263 | 2.74 | 11.75 | 60 | 0.42 |
| N=18 | 531±598 | 1.54±1.25 | 8.88±4.30 | 75±41 | 0.62±0.28 |
