# Supplementary material for: A comparison of neuronal population dynamics measured with calcium imaging and electrophysiology
Source: PLoS Comput Biol. 2020 Sep 15;16(9):e1008198. doi: 10.1371/journal.pcbi.1008198 (PMC7518847; doi:10.1371/journal.pcbi.1008198)
Supplement: Attachment — Submitted filename: Response_to_reviewers_Wei_et_al_v9.docx [file pcbi.1008198.s011.docx]

Reviewer #1: Calcium imaging has become a common tool for measuring the selectivity and dynamics of neuronal activity in response to stimulus features, task events and motor output. However, while it is widely appreciated that the fluorescence signals measured with calcium imaging are not a direct report of spiking activity, the set of potential errors introduced by these indirect measurements, and the sources of these errors, have not been thoroughly elucidated. In the current manuscript the authors take advantage of existing datasets in which they collected either electrophysiology or calcium imaging data during the same behavioral task. When comparing these datasets, the authors found striking differences in the representation of trial-type selectivity in the population that could not be explained by differences in the depths of the recorded populations or errors in spike-sorting, and could not be corrected by using standard spike extraction methods. To understand the source of these errors, the authors then made simultaneous imaging and electrophysiological recordings from dozens of neurons to generate a forward model that enabled direct transformation of spikes to fluorescence. This model revealed that the specific parameters of the transformation could not simply be accounted for by the indicator used and must instead be tuned on a cell-by-cell basis to generate a good prediction. Moreover, using this forward model to generate synthetic fluorescence traces from spike trains, the authors could recapitulate the differences seen in the imaging and electrophysiology datasets, suggesting that the spike to fluorescence transformation could explain the errors. The authors then proceeded to explore a number of standard analytical approaches commonly used to measure the temporal dynamics and task selectivity of neuronal populations and reveal the fundamental errors introduced by the spike to fluorescence transformation.

This is a rigorous and careful study that reveals systematic errors that calcium imaging can introduce. These results will aid in the careful interpretation of future experiments, and reinterpretation of existing ones (Figure 7 was particularly dramatic in this regard), especially when considering the temporal dynamics and complex selectivity of neuronal populations. Overall, the manuscript is well-written and the complex datasets and analyses are clearly illustrated. I have only a few minor suggestions to improve the clarity of the manuscript.

1. While all of the motivation and analyses will be clear to readers that are familiar with common approaches used in systems neuroscience, some sections are rather terse and could use some additional explanation. For instance, the results describing Figure 3 are described in three sentences (Lines 149-153). Additionally, more background on why the specific analytical examples were chosen (i.e. PCA, decoding, and distribution of peak fluorescence times) and how these are typically interpreted would be useful for readers that are newer to the field.

We thank the reviewer for this suggestion. We extended the description of the results in Figure 3 and added sentences clarifying motivations for the population analyses. Part of the reason that the results of Figure 3 were described briefly is that the data of figure 3 becomes the basis for the spike to fluorescence modeling described in Figure 4 and beyond. We now both added a longer description and added a sentence clarifying this point. In addition, we added clarifying sentences for all population analyses and a supplementary figure regarding dispersal of dynamics. For ease of reference we copied the text motivating each analysis and mark in italics the new added text.

**Simultaneous recordings:** *Peak fluorescence responses increased monotonically with the number of spikes.* The ability to detect single spikes varied considerably between neurons (Figures 3E-G, S2). The detection of single spikes was lower in transgenic mice than with AAV-based gene transduction, likely reflecting the lower expression level in the transgenic mice. *Detection of spikes varied across neurons and, as expected, was better for 6s-TG than 6f-TG (****Figure 3F****). We used these simultaneous recordings to build models of the spike to fluorescence transformation.*

**PCA:** Large-scale recording methods are often used in combination with dimensionality reduction techniques to provide a compact description of the data(30). For example, principal component analysis (PCA) finds modes of population activity that capture the largest amount of variance in neural activity(30). Data visualization and analysis are often performed after truncating the decomposition after a few components. *Moreover, regression analyses are typically performed following dimensionality reduction to avoid having the number of variables (neurons) be close to the number of samples (trials).*

**Decoding:** Decoding analysis relating population activity to behavioral variables is widely used in systems neuroscience(3, 6, 33, 51). Such analyses typically relate the state of population activity at a given time point to a behavioral variable of interest, such as behavioral choice. *They are one of the most common analyses as they are a form of addressing the question of what information does a certain population of neurons contain.*

**Distribution of peak fluorescence:** Neurons show temporally complex responses, even in simple trial-based behaviors(25, 26). These spike rate changes are critical for an understanding of neural circuit models of neural computation. *For instance, relative timing can be analyzed for evidence of propagation of sequential propagation of information.*

2. The authors show that the inferred firing rates cannot “rescue” the differences between calcium imaging and electrophysiology for the distribution of tuning types (Figure 2) or temporal dispersion (Figure 7). It would be helpful to include these analyses for Figures 5 and 6 in the main figures rather than in the supplement so that they can be directly compared.

We thank the reviewer for this suggestion. We now moved the material from supplementary figures S5 and S6 into the main figures, Figures 5 and 6.

We include the new figures here for convenience:

Figure 5

Figure 6:

3. Why are the values in Figure 3G so similar between 6f and 6s when they’re so different for 3D and 3F?

We now see the issue and we believe the organization of the plots may have accidentally caused this impression. First, just to clarify, Figure 3D shows two example neurons and single dots are individual spiking events whereas Figure 3G overlays all neurons, and each dot is a neuron. We put in Fig. 3G to show the scaling of detectability beyond single spike events. However, since we showed just peak deltaF/F and not the noise, it made it hard to compare to 3F, which we agree becomes confusing. We experimented with different ways to make the point clearer and now added a zero AP column, lines connecting neurons and more AP values. We copy the figure here for ease of reference:

4. Is the forward model for 6f-TG significantly worse than for the other indicators? If so, how should this be interpreted?

The mean variance explained is indeed slightly smaller in our sample, but it does not reach statistical significance (p=.0614, Kruskal-Wallis ANOVA Test). In 6f-TG there is both increased background noise and variability in the responses associated with spiking events, both of which contribute to lower variance explained.

Typos
1. Line 317- “have one or the either”

2. Line 319- “These allow to use or analyses and models…”

3. Line 413- “(insert panels)”

We thank the reviewer for pointing out these typos. We have corrected them in the text.

Reviewer #2: It will be no surprise to many neuroscientists that calcium imaging and electrophysiology present different views of neuronal population dynamics. And yet I can think of no example as striking as this paper by Wei et al. who present a rigorous and detailed study of neural dynamics in mice performing a whisker object-location task. They illustrate how the slow dynamics of calcium measurements can obscure the timing of spikes, resulting in misclassification of neurons as non-selective for trial type. The temporal dispersion affects decoding and dimensionality reduction by PCA and none of these problems are recovered by spike inference. Although complex, the results and analyses are presented with absolute clarity. Papers that illustrate and explore a problem are often influential and I expect this paper will be no exception.

I have only one suggestion. Around line 120 the authors compare spike inference algorithms. How did they set the parameters for each model? Is it possible that the failure of the models to produce spike-like numbers of multiphasic neurons is due to mis-tuning of the models?

We completely agree with the reviewer that one of the issues when performing algorithm comparison is that published approaches may rely on parameters that are difficult to tune. Accordingly, we intentionally selected fluorescence-to-spike models that do not require manual setting of parameters or hyperparameters. Instead, these toolboxes autonomously fit the parameters they need through optimization processes provided in the shared code. We have now added a sentence in the methods section clarifying this point. We note in addition that we verified and ensured that fluorescence-to-spike models were implemented correctly and return reasonable results with their automated parameter settings by examining the simultaneous recorded loose patch and imaging data (where the loose patch provides ground truth).

Reviewer #3: We apologize to the authors and editors for the tardy review.

The Wei et al. manuscript will make an important contribution to the field. It is well motivated, addresses specific open questions, and will serve as a guide (and cautionary tale) for scientists seeking to perform calcium imaging recordings. In particular, we see the strengths of this work to be twofold: 1) the analysis of low-dimensional population activity demonstrating qualitative differences between conclusions drawn from electrophysiological records and calcium imaging; and 2) the demonstration that deconvolution algorithms still struggle to ‘undo’ the spike-to-fluorescence transformation in a robust way. This study contains the clearest and most informative demonstration to date of how the properties of calcium imaging perturb the conclusions that can be drawn about the tuning properties and firing time courses of neurons.

Although we enthusiastically support publication of this study, there are several concerns that should be addressed.

Major concerns

Quality control of ROIs: The authors do not discuss criteria used for including ROIs in their datasets. Forgive us for this speculative criticism, but we want to be sure that the major effects described in this work are not an artefact of including low quality ROIs. A simple but clear description of the inclusion criteria (and consequently, how much data was thrown out) of each dataset is needed. Ideally, a more thorough treatment would reanalyze the data sets and parameterize the perturbations with respect to a sliding quality metric (like SNR). e.g. Do high SNR neurons have a higher percentage of down ramping neurons? The quality control metric can be applied at two stages. First, the fluorescence transient derived from analysis of each ROI should be analyzed - how much neuropil contamination is there, what is its SNR, how dim is it (how many photons contribute to the signal), etc. Second, it is also imperative to do these analyses as a function of the quality metric used to define an ROI as a cell in the first place. This might be PC score of the pixels, another covariance related metric, or perhaps a more heuristic score considering many variables. The score should reflect the likelihood of experimenter including that ROI and the fluorescence extracted from it in a dataset.

(see [https://www.sciencedirect.com/science/article/pii/S0959438818300977](https://urldefense.com/v3/__https:/www.sciencedirect.com/science/article/pii/S0959438818300977__;!!Eh6p8Q!UZ3CIft2ETM940gLge8Oqx_RKcZmyy0M-U4p7JltDTS_l4zc-XVzf121v0NXc-LXyw$))

We thank the reviewers for suggestions regarding quality control of ROIs. In all these datasets ROIs were selected manually and hence did not strictly have a precise quality control metric that determined their inclusion. We also note that some of these datasets are associated with published papers and we did not change the preprocessing used. As a first step, we added the detailed information of the manual ROI selection in the Methods sections to make sure the ROI selection and preprocessing would be clear to readers.

As a second step we followed the reviewer’s suggestion for a sliding threshold SNR analysis. We estimated SNR using the procedure in the widely used CaImAn package, in which the noise level is estimated as the exponential of the mean of the logarithm of power spectral density (we checked that suite2p uses the same algorithm for noise estimation). We then generated datasets including only a subset of neurons by moving the threshold up from its zeroth percentile to its 100^th^ percentile. We added this figure as supplementary figure and reproduce it here for ease of reference:

.

As can be seen, for instance for 6s-AAV across no value of SNR threshold did the main differences, the lower frequency of multi-selective neurons, the higher number of contra preferring neurons or the relative fraction of ramping up and down neurons change substantially (we remind the reviewer that 6f data due to its faster dynamics has a contra-ipsi ratio of close to half like ephys). The number of selective neurons of course changed since the way that SNR is calculated involves deviations from average activity which is naturally correlated partially with selectivity as one cannot get selectivity without deviations from average activity. For this reason, we are wary of changing results based on thresholding on SNR. Such preselection needs to be done with care in order not to introduce confounds. Though we appreciate the more general suggestion of switching to automated ROI detection, we note that automated ROI detection can also have its own issues and sensitivities to artefacts. A full reanalysis of the primary data based on one (of many) automated approaches and a detailed comparison to the manual ROI selection is beyond the scope of this paper, and since the datasets were collected and published with manual ROI selection, we kept the neuron selection as originally chosen. To highlight this potential issue to the reader we added the figure above as a supplementary figure to the paper and referenced the issue of low SNR ROIs.

Fig 4B shows that for some parameters, there is non-zero probability that some neurons have parameters that seem impossible (eg tau_d of zero, or c_1/2 of zero). This suggests to us that many ROIs that shouldn’t pass quality control are being included in this loose patch dataset. A comment about the quality control used to restrict the analysis of this data is necessary, especially given the difficulty of these types of recordings.

We thank the reviewer for pointing out this potentially confusing issue. The seemingly unreasonable model parameters are not due to some issue with quality control, but are rather just an artefact of visualization. As we wanted to present on one plot multiple distributions we used a smoothing procedure to allow for better visualization (rather than overlaying bars in the exact same bins). The smoothed curves can then take values that are less reasonable than the actual data. We note however that Figure S3 in the original submission showed the same data as scatter plots and of course no neurons have tau_d_ or c_1/2_ values of zero. For ease of reference we also present here the non-smoothed histograms. Note also that we are confident the recorded neurons were good ROIs by manual inspection of the high-zoom imaging video and the simultaneous loose-cell recordings.

Depth control of ROIs: Related the 1), deeper ROIs tend to be noisier due to optical constraints, and layer 5 cells tend to have different calcium buffering properties that may make their response more non-linear.
In the second paragraph, the authors describe the details of the primary datasets used. It would be very surprising if the numbers of ROIs collected are from single imaging sessions. While it is common to extract greater than 1500 neurons using transgenic animals and a large FOV objective, it is also typical to then throw away 1/4 to 3/4 of those ROIs by imposing inclusion criteria. These criteria can be very strict when attempting to examine fine temporal dynamics or perform deconvolution.

The number of cells reported were indeed not from single imaging sessions. We thank the reviewer for pointing out this potential source of confusion. We now list in the Materials and Methods the average number of neurons per session. In addition, we plot below the full distribution of neuron numbers per session.

Inclusion of OASIS: We were surprised that although 2 deconvolution methods were tested (and 7 were mentioned), OASIS was not included. We have no stake in OASIS, but to our knowledge it is the most commonly used method, owing partly to it’s inclusion in the Suite2p package.

We apologize for the confusion here. We used the CalmAn package which solves the FOOPSI problem to infer firing rates and then uses MCMC to infer spike times. For the firing rate step the package uses OASIS as its default option (see follow link as a reference).

<https://github.com/flatironinstitute/CaImAn/blob/3683b8726d1cc8e553afd88b62e4d82cf931ed4b/caiman/source_extraction/cnmf/deconvolution.py#L23>

We clarified this point in the methods section.

Moderate concerns

Having an N=1 for the Thy1-GCamp6s(4.3) condition is a little concerning for generalizing the interpretation of these results. We understand that asking to expand this may be a big request, but it would greatly strengthen our confidence in the results if additional data can be included. Or, if using quality control metrics, we are reassured that this dataset is of the same quality as the others. Of course, it is problematic to infer much about population distributions from N=1.

We agree with the reviewer that ideally more data could be added. However, in the current state of experimental work due to the covid-19 response that was unfortunately not feasible. We note that all results on this data are qualitatively consistent with the 6s-AAV data and we therefore elt that keeping this data is a more sensible choice than entirely removing it from the paper. To make sure readers are aware of this issue we added a sentence immediately following the introduction of the dataset:

“We note that the 6s-TG data, though containing a large number of neurons across multiple behavioral sessions, came from a single animal”. We then clarify in more depth in the methods section: “Though we find this data to be consistent with other imaging data, the single animal source could be a potential issue in that differences in an individual animal’s behavior may cause differences in neural encoding.”

Strawmanning: While this is not a major issue, we fear that the authors may be ‘begging the question’ slightly by comparing raw dF/F traces against ephys traces in a task that involves epochs on the timescales of the kinetics of the calcium indicators used. It is known that deconvolution struggles to resolve exact spike timing when there is noise in the trace. It is therefore critical that the conclusions stated here emphasize the fact that the qualitative differences between ephys and calcium imaging exist on short-time scales only. That is to say the degree to which one can reasonably expect to be able to resolve relevant temporal dynamics is dependent on the overlap of the frequency content of the fluorescent reporter and the underlying dynamics, as well as the signal-to-noise and sampling rate of the recording. All four of these parameters come together to determine whether inference or reconstruction is possible. This paper does a remarkable job of illustrating how things can go wrong, but it may do a bit of disservice to the field to not also demarcate acceptable boundaries for which calcium imaging fairly approximates underlying dynamics. This might be addressed by something as simple as a paragraph defining what the authors see as acceptable signal-to-noise and sampling rate necessary to use for their task, and/or the minimum inter-epoch interval appropriate to use for common calcium imaging data.
The range of depths they use is very wide. There are differences in biophysical properties of neurons (like ca buffering) and optical differences (hitting the noise floor) between 100-800µm. These differences result in nonlinear changes in the estimation of the amplitude of transients and deconvolved event rates. Indeed, the authors observe very significant differences in the coding properties of neurons imaged at different depths. However, the observed differences don’t follow similar trends, and it is difficult to use the hypothesized model to account for these depth dependent differences. The authors do not adequately address the coding differences seen across depths.

We completely agree with the reviewer regarding the points that the reviewer raises and very much agree with the elegant way they put it as follows: “That is to say the degree to which one can reasonably expect to be able to resolve relevant temporal dynamics is dependent on the overlap of the frequency content of the fluorescent reporter and the underlying dynamics, as well as the signal-to-noise and sampling rate of the recording”. The main difficulty is that the relevant temporal dynamics in any brain area are heterogenous and the type and degree of heterogeneity may vary from area to area. Moreover, the heterogeneity of biophysics relevant to Ca dynamics may vary from area to area and from neuron type to neuron type. Similarly, the processing by the indicator itself may be variable. Finally, the metrics used in a study may be more sensitive to different frequency components. Indeed, we feel one of the main contributions of this paper is highlighting these interactions and we have two lengthy sections in the discussion section talking about these points (which we copy below for ease of reference). Our most informed opinion, as we explain in these paragraphs, is that we cannot just assume a generic formula that will hold for all questions, areas and conditions. Rather one should directly investigate specifics for a given area and hypothesis with ephys recordings and forward models. Indeed, this is why we created *im-phys.org* precisely since we cannot encapsulate the subject in a single paragraph but still what to enable informed decisions.

From the discussion:

*“****Going forward***

*Going forward, the discrepancies between ephys and calcium imaging can be reduced by improvements in calcium indicators, adjustments to experimental design and use of forward-models to identify the sensitivity of metrics if interest to the transformation in calcium dynamics. Calcium indicators could be improved on multiple fronts. They could be made faster and less nonlinear (18, 62). In addition, more uniform expression across cells can allow for more aggressive modeling of the nonlinearities that cannot be reduced, especially when coupled with priors on activity profiles derived from large scale electrophysiology. Faster indicators will result in the effect of previous activity history washing away faster, thus reducing effects that are history dependent. Imaging with multiple types of indicators in different experiments might produce additional constraints and help reduce biases. Voltage imaging holds great potential for fast accurate measurement of spiking activity, at least in sparsely labeled neuronal populations (63, 64). At the level of experimental design, when population activity in a given behavioral epoch involves fixed dynamics, such as settling to a steady state or consistent ramping, longer trial epochs will allow the effect of the previous dynamical state to decay away. Indeed, we found a smaller discrepancy between the number of multiphasic neurons in ephys and 6s-TG data when the behavioral paradigm was adapted to use longer delay epochs.*

*Finally and most importantly, the sensitivity to the specific properties of population activity that are of interest to a particular hypothesis can be evaluated by forward models, as we performed here. For example, imaging studies could use forward models on published ephys data to evaluate the potential effect of the spike to calcium transformation on the metrics of interest. Then differences from the expected value given the transformation can be analyzed and metrics that are shown to be more variable given heterogeneity in transformation parameters can be flagged. This effort will become easier as neurophysiology probes become more powerful (65), data sharing more common, and preprocessing more standardized”*

Explanation of intracellular recordings is very poor. This is important data. I can’t find anywhere in the referenced paper where the data is from (Guo et al 2017) intracellular recordings made during the behavior, only optogenetics. This data should be referenced and described when introducing the other ephys and imaging datasets.

The data were from Guo et al., 2017 (total N=36 cells in 3 different experiment conditions, where we excluded one cell due to the small number of trials). The data are described in Figure 4 and Extended data figures 5,6. We note that the confusion with the photoinhibition may be that the (very succinct) legends of figure 4 describe the data mostly with respect to the photoinhibition results, but photoibhibition wasn’t performed on each trial and we only applied the analyses to the data using the trials without photostimulation. We now added a clearer description of intracellular data from Guo et al., 2017 to the Method section.

Minor concerns

Textual concern: The intro makes it sound like they are simultaneously recording the same neurons.

Unexplored and unaddressed is whether gcamp expression itself affects tuning properties.

We thank the reviewer for this comment. We changed the text of the intro. From “We first analyzed ephys and calcium imaging data measured in matched neuronal populations” to “We first analyzed ephys and calcium imaging data recorded separately but measured in matched neuronal populations”

Fig 2H is a bit strange. The number of neurons classified as mono OR multiphasic is less than the number classified as mono or multiphasic using ephys. So, what are all those neurons classified as? Nonselective? Please show several example traces of what happens to a neuron that was classified as monophasic selective before deconvolution and then not monoselective (or specifically nonselective) after deconvolution. Please also show traces of imaging traces going from nonselective to multiphasic.

We added example neurons that changed from being monoselective to nonselective or multiselective after deconvolution (new Figure S4D) and we plot the transition matrix, i.e., grouping data separately by their selectivity class in the original imaging data, we show what fraction is found in each selectivity class after spike inference (new Figure S4E). We reproduce Figure S4D-E here for ease of reference:

Typo line 143: ‘simultaneous’

We thank the reviewer for pointing out the typo. We have revised it in the text.

The example trace in 4A doesn’t seem that representative. Average EV was around 85%.

We added a trace from a neuron that had 85% explained variance as Figure 4F.

Shouldn’t fig 4c/d top be able to transform into 4c/d bottom by convolution with the determined kernel of that neuron?

We apologize if this was unclear, this is exactly what we did. We clarified this point in the text.

Please provide additional comment on analysis methods used to break down the variance contained in particular principle components

The breakdown of variance is given in equations 4 and 5. We have added a direct note that those are the relevant equations in the text.

The following paper should be mentioned/contrasted: Robustness of Spike Deconvolution for Neuronal Calcium Imaging by Marius Pachitariu, Carsen Stringer and Kenneth D. Harris

We thank the reviewer for this suggestion and now reference this paper. We feel that the papers are very different in focus. The paper mentioned by the reviewer benchmarks several spike inference algorithms and its main significance, as defined by the authors, is in showing that more sophisticated supervised learning algorithms do not outperform simpler approaches. To make sure we are not misrepresenting that paper, here is the significance statement of that paper: *“The experimental method that currently allows for recordings of the largest numbers of cells simultaneously is two-photon calcium imaging. However, use of this powerful method requires that neuronal firing times be inferred correctly from the large resulting datasets. Previous studies have claimed that complex supervised learning algorithms outperform simple deconvolution methods at this task. Unfortunately, these studies suffered from several problems and biases. When we repeated the analysis, using the same data and correcting these problems, we found that simpler spike inference methods perform better. Even more importantly, we found that supervised learning methods can introduce artifactual structure into spike trains, that can in turn lead to erroneous scientific conclusions. Of the algorithms we evaluated, we found that an extremely simple method performed best in all circumstances tested, was much faster to run, and was insensitive to parameter choices, making incorrect scientific conclusions much less likely.”*

Following the approach presented in the paper suggested by the reviewer we applied the binned spearman correlation measure as in Pachitariu et al. to examine the spike inference models on our simultaneous recordings. In agreement with Pachitariu et al. we find that complex models do not consistently give better performance than simpler models (e.g., MLSpike is outperformed by the nonnegative wiener filter approach) yet the MCMC model, which we used in the manuscript, had the best performance overall. This strengthens our conclusions, as even the best performing spike inference model according to this metric as well did not fully undo the differences we find.

Please comment on why the spike inference data in S6 saturates at lower levels than the raw ephys decoder. Also please comment on the peaky shape of the MLSpike decoder accuracy over time? Can these issues be resolved by convolving the deconvolved data with wider gaussian kernels? Is it fair to assume that the spike inference decoders are convolved with the same smoothing kernels as the ephys decoders?

We believe it is hard to say a priori whether and by how much spike inference should change decoding accuracy. We can comment however that these results are consistent with the single-neuron selectivity analyses in Figure 2, in that spike inference reduces the fraction of selective neurons (and to a level that is less than in ephys), which is consistent with a decrease of decodability.

The spiky decoding accuracy is due to MLSpike’s tendency to generate spiky firing rates. This might indeed be resolved by applying a wide gaussian kernel. However, that might have other effects, and importantly we did not apply smoothing kernels on the ephys decoders.

Overall, an excellent paper. Well done.

Thank you!
