# Supplementary material for: A comparison of neuronal population dynamics measured with calcium imaging and electrophysiology
Source: PLoS Comput Biol. 2020 Sep 15;16(9):e1008198. doi: 10.1371/journal.pcbi.1008198 (PMC7518847; doi:10.1371/journal.pcbi.1008198)
Supplement: Peer review history

# Peer review: Decision Letter 0

**DOI:** 10.1371/journal.pcbi.1008198.r001

**Authors:** Boris S. Gutkin, Kim T. Blackwell

13 May 2020

Dear Prof. Druckmann,

Thank you very much for submitting your manuscript "A comparison of neuronal population dynamics measured with calcium imaging and electrophysiology" for consideration at PLOS Computational Biology.

As with all papers reviewed by the journal, your manuscript was reviewed by members of the editorial board and by several independent reviewers. In light of the reviews (below this email), we would like to invite the resubmission of a significantly-revised version that takes into account the reviewers' comments. We would like the authors to pay particular attention to the major issues brought up by the reviewer 3.

We cannot make any decision about publication until we have seen the revised manuscript and your response to the reviewers' comments. Your revised manuscript is also likely to be sent to reviewers for further evaluation.

When you are ready to resubmit, please upload the following:

[1] A letter containing a detailed list of your responses to the review comments and a description of the changes you have made in the manuscript. Please note while forming your response, if your article is accepted, you may have the opportunity to make the peer review history publicly available. The record will include editor decision letters (with reviews) and your responses to reviewer comments. If eligible, we will contact you to opt in or out.

[2] Two versions of the revised manuscript: one with either highlights or tracked changes denoting where the text has been changed; the other a clean version (uploaded as the manuscript file).

Important additional instructions are given below your reviewer comments.

Please prepare and submit your revised manuscript within 60 days. If you anticipate any delay, please let us know the expected resubmission date by replying to this email. Please note that revised manuscripts received after the 60-day due date may require evaluation and peer review similar to newly submitted manuscripts.

Thank you again for your submission. We hope that our editorial process has been constructive so far, and we welcome your feedback at any time. Please don't hesitate to contact us if you have any questions or comments.

Sincerely,

Boris S. Gutkin

Associate Editor

PLOS Computational Biology

Kim Blackwell

Deputy Editor

PLOS Computational Biology

***********************

Reviewer's Responses to Questions

**Comments to the Authors:**

**Please note here if the review is uploaded as an attachment.**

Reviewer #1: Calcium imaging has become a common tool for measuring the selectivity and dynamics of neuronal activity in response to stimulus features, task events and motor output. However, while it is widely appreciated that the fluorescence signals measured with calcium imaging are not a direct report of spiking activity, the set of potential errors introduced by these indirect measurements, and the sources of these errors, have not been thoroughly elucidated. In the current manuscript the authors take advantage of existing datasets in which they collected either electrophysiology or calcium imaging data during the same behavioral task. When comparing these datasets, the authors found striking differences in the representation of trial-type selectivity in the population that could not be explained by differences in the depths of the recorded populations or errors in spike-sorting, and could not be corrected by using standard spike extraction methods. To understand the source of these errors, the authors then made simultaneous imaging and electrophysiological recordings from dozens of neurons to generate a forward model that enabled direct transformation of spikes to fluorescence. This model revealed that the specific parameters of the transformation could not simply be accounted for by the indicator used and must instead be tuned on a cell-by-cell basis to generate a good prediction. Moreover, using this forward model to generate synthetic fluorescence traces from spike trains, the authors could recapitulate the differences seen in the imaging and electrophysiology datasets, suggesting that the spike to fluorescence transformation could explain the errors. The authors then proceeded to explore a number of standard analytical approaches commonly used to measure the temporal dynamics and task selectivity of neuronal populations and reveal the fundamental errors introduced by the spike to fluorescence transformation.

This is a rigorous and careful study that reveals systematic errors that calcium imaging can introduce. These results will aid in the careful interpretation of future experiments, and reinterpretation of existing ones (Figure 7 was particularly dramatic in this regard), especially when considering the temporal dynamics and complex selectivity of neuronal populations. Overall, the manuscript is well-written and the complex datasets and analyses are clearly illustrated. I have only a few minor suggestions to improve the clarity of the manuscript.

1. While all of the motivation and analyses will be clear to readers that are familiar with common approaches used in systems neuroscience, some sections are rather terse and could use some additional explanation. For instance, the results describing Figure 3 are described in three sentences (Lines 149-153). Additionally, more background on why the specific analytical examples were chosen (i.e. PCA, decoding, and distribution of peak fluorescence times) and how these are typically interpreted would be useful for readers that are newer to the field.

2. The authors show that the inferred firing rates cannot “rescue” the differences between calcium imaging and electrophysiology for the distribution of tuning types (Figure 2) or temporal dispersion (Figure 7). It would be helpful to include these analyses for Figures 5 and 6 in the main figures rather than in the supplement so that they can be directly compared.

3. Why are the values in Figure 3G so similar between 6f and 6s when they’re so different for 3D and 3F?

4. Is the forward model for 6f-TG significantly worse than for the other indicators? If so, how should this be interpreted?

Typos

1. Line 317- “have one or the either”

2. Line 319- “These allow to use or analyses and models…”

3. Line 413- “(insert panels)”

Reviewer #2: It will be no surprise to many neuroscientists that calcium imaging and electrophysiology present different views of neuronal population dynamics. And yet I can think of no example as striking as this paper by Wei et al. who present a rigorous and detailed study of neural dynamics in mice performing a whisker object-location task. They illustrate how the slow dynamics of calcium measurements can obscure the timing of spikes, resulting in misclassification of neurons as non-selective for trial type. The temporal dispersion affects decoding and dimensionality reduction by PCA and none of these problems are recovered by spike inference. Although complex, the results and analyses are presented with absolute clarity. Papers that illustrate and explore a problem are often influential and I expect this paper will be no exception.

I have only one suggestion. Around line 120 the authors compare spike inference algorithms. How did they set the parameters for each model? Is it possible that the failure of the models to produce spike-like numbers of multiphasic neurons is due to mis-tuning of the models?

Reviewer #3: We apologize to the authors and editors for the tardy review.

The Wei et al. manuscript will make an important contribution to the field. It is well motivated, addresses specific open questions, and will serve as a guide (and cautionary tale) for scientists seeking to perform calcium imaging recordings. In particular, we see the strengths of this work to be twofold: 1) the analysis of low-dimensional population activity demonstrating qualitative differences between conclusions drawn from electrophysiological records and calcium imaging; and 2) the demonstration that deconvolution algorithms still struggle to ‘undo’ the spike-to-fluorescence transformation in a robust way. This study contains the clearest and most informative demonstration to date of how the properties of calcium imaging perturb the conclusions that can be drawn about the tuning properties and firing time courses of neurons.

Although we enthusiastically support publication of this study, there are several concerns that should be addressed.

Major concerns

Quality control of ROIs: The authors do not discuss criteria used for including ROIs in their datasets. Forgive us for this speculative criticism, but we want to be sure that the major effects described in this work are not an artefact of including low quality ROIs. A simple but clear description of the inclusion criteria (and consequently, how much data was thrown out) of each dataset is needed. Ideally, a more thorough treatment would reanalyze the data sets and parameterize the perturbations with respect to a sliding quality metric (like SNR). e.g. Do high SNR neurons have a higher percentage of down ramping neurons? The quality control metric can be applied at two stages. First, the fluorescence transient derived from analysis of each ROI should be analyzed - how much neuropil contamination is there, what is its SNR, how dim is it (how many photons contribute to the signal), etc. Second, it is also imperative to do these analyses as a function of the quality metric used to define an ROI as a cell in the first place. This might be PC score of the pixels, another covariance related metric, or perhaps a more heuristic score considering many variables. The score should reflect the likelihood of experimenter including that ROI and the fluorescence extracted from it in a dataset.

(see https://www.sciencedirect.com/science/article/pii/S0959438818300977)

Fig 4B shows that for some parameters, there is non-zero probability that some neurons have parameters that seem impossible (eg tau_d of zero, or c_1/2 of zero). This suggests to us that many ROIs that shouldn’t pass quality control are being included in this loose patch dataset. A comment about the quality control used to restrict the analysis of this data is necessary, especially given the difficulty of these types of recordings.

Depth control of ROIs: Related the 1), deeper ROIs tend to be noisier due to optical constraints, and layer 5 cells tend to have different calcium buffering properties that may make their response more non-linear.

In the second paragraph, the authors describe the details of the primary datasets used. It would be very surprising if the numbers of ROIs collected are from single imaging sessions. While it is common to extract greater than 1500 neurons using transgenic animals and a large FOV objective, it is also typical to then throw away 1/4 to 3/4 of those ROIs by imposing inclusion criteria. These criteria can be very strict when attempting to examine fine temporal dynamics or perform deconvolution.

Inclusion of OASIS: We were surprised that although 2 deconvolution methods were tested (and 7 were mentioned), OASIS was not included. We have no stake in OASIS, but to our knowledge it is the most commonly used method, owing partly to it’s inclusion in the Suite2p package.

Moderate concerns

Having an N=1 for the Thy1-GCamp6s(4.3) condition is a little concerning for generalizing the interpretation of these results. We understand that asking to expand this may be a big request, but it would greatly strengthen our confidence in the results if additional data can be included. Or, if using quality control metrics, we are reassured that this dataset is of the same quality as the others. Of course, it is problematic to infer much about population distributions from N=1.

Strawmanning: While this is not a major issue, we fear that the authors may be ‘begging the question’ slightly by comparing raw dF/F traces against ephys traces in a task that involves epochs on the timescales of the kinetics of the calcium indicators used. It is known that deconvolution struggles to resolve exact spike timing when there is noise in the trace. It is therefore critical that the conclusions stated here emphasize the fact that the qualitative differences between ephys and calcium imaging exist on short-time scales only. That is to say the degree to which one can reasonably expect to be able to resolve relevant temporal dynamics is dependent on the overlap of the frequency content of the fluorescent reporter and the underlying dynamics, as well as the signal-to-noise and sampling rate of the recording. All four of these parameters come together to determine whether inference or reconstruction is possible. This paper does a remarkable job of illustrating how things can go wrong, but it may do a bit of disservice to the field to not also demarcate acceptable boundaries for which calcium imaging fairly approximates underlying dynamics. This might be addressed by something as simple as a paragraph defining what the authors see as acceptable signal-to-noise and sampling rate necessary to use for their task, and/or the minimum inter-epoch interval appropriate to use for common calcium imaging data.

The range of depths they use is very wide. There are differences in biophysical properties of neurons (like ca buffering) and optical differences (hitting the noise floor) between 100-800µm. These differences result in nonlinear changes in the estimation of the amplitude of transients and deconvolved event rates. Indeed, the authors observe very significant differences in the coding properties of neurons imaged at different depths. However, the observed differences don’t follow similar trends, and it is difficult to use the hypothesized model to account for these depth dependent differences. The authors do not adequately address the coding differences seen across depths.

Explanation of intracellular recordings is very poor. This is important data. I can’t find anywhere in the referenced paper where the data is from (Guo et al 2017) intracellular recordings made during the behavior, only optogenetics. This data should be referenced and described when introducing the other ephys and imaging datasets.

Minor concerns

Textual concern: The intro makes it sound like they are simultaneously recording the same neurons.

Unexplored and unaddressed is whether gcamp expression itself affects tuning properties.

Fig 2H is a bit strange. The number of neurons classified as mono OR multiphasic is less than the number classified as mono or multiphasic using ephys. So, what are all those neurons classified as? Nonselective? Please show several example traces of what happens to a neuron that was classified as monophasic selective before deconvolution and then not monoselective (or specifically nonselective) after deconvolution. Please also show traces of imaging traces going from nonselective to multiphasic.

Typo line 143: ‘simultaneous’

The example trace in 4A doesn’t seem that representative. Average EV was around 85%.

Shouldn’t fig 4c/d top be able to transform into 4c/d bottom by convolution with the determined kernel of that neuron?

Please provide additional comment on analysis methods used to break down the variance contained in particular principle components

The following paper should be mentioned/contrasted: Robustness of Spike Deconvolution for Neuronal Calcium Imaging by Marius Pachitariu, Carsen Stringer and Kenneth D. Harris

Please comment on why the spike inference data in S6 saturates at lower levels than the raw ephys decoder. Also please comment on the peaky shape of the MLSpike decoder accuracy over time? Can these issues be resolved by convolving the deconvolved data with wider gaussian kernels? Is it fair to assume that the spike inference decoders are convolved with the same smoothing kernels as the ephys decoders?

Overall, an excellent paper. Well done.

**********

**Have all data underlying the figures and results presented in the manuscript been provided?**

Large-scale datasets should be made available via a public repository as described in the *PLOS Computational Biology* data availability policy, and numerical data that underlies graphs or summary statistics should be provided in spreadsheet form as supporting information.

Reviewer #1: Yes

Reviewer #2: Yes

Reviewer #3: Yes

**********

PLOS authors have the option to publish the peer review history of their article (what does this mean?). If published, this will include your full peer review and any attached files.

If you choose “no”, your identity will remain anonymous but your review may still be made public.

**Do you want your identity to be public for this peer review?** For information about this choice, including consent withdrawal, please see our Privacy Policy.

Reviewer #1: No

Reviewer #2: No

Reviewer #3: No

Figure Files:

While revising your submission, please upload your figure files to the Preflight Analysis and Conversion Engine (PACE) digital diagnostic tool, https://pacev2.apexcovantage.com. PACE helps ensure that figures meet PLOS requirements. To use PACE, you must first register as a user. Then, login and navigate to the UPLOAD tab, where you will find detailed instructions on how to use the tool. If you encounter any issues or have any questions when using PACE, please email us at figures@plos.org.

Data Requirements:

Please note that, as a condition of publication, PLOS' data policy requires that you make available all data used to draw the conclusions outlined in your manuscript. Data must be deposited in an appropriate repository, included within the body of the manuscript, or uploaded as supporting information. This includes all numerical values that were used to generate graphs, histograms etc.. For an example in PLOS Biology see here: http://www.plosbiology.org/article/info%3Adoi%2F10.1371%2Fjournal.pbio.1001908#s5.

Reproducibility:

To enhance the reproducibility of your results, PLOS recommends that you deposit laboratory protocols in protocols.io, where a protocol can be assigned its own identifier (DOI) such that it can be cited independently in the future. For instructions, please see http://journals.plos.org/compbiol/s/submission-guidelines#loc-materials-and-methods

# Author comment: Author response to Decision Letter 0

**DOI:** 10.1371/journal.pcbi.1008198.r002

20 Jul 2020

Attachment Submitted filename: Response_to_reviewers_Wei_et_al_v9.docx Click here for additional data file.

# Editor report: Decision Letter 1

**DOI:** 10.1371/journal.pcbi.1008198.r003

**Authors:** Boris S. Gutkin, Kim T. Blackwell

27 Jul 2020

Dear Prof. Druckmann,

We are pleased to inform you that your manuscript 'A comparison of neuronal population dynamics measured with calcium imaging and electrophysiology' has been provisionally accepted for publication in PLOS Computational Biology.

Before your manuscript can be formally accepted you will need to complete some formatting changes, which you will receive in a follow up email. A member of our team will be in touch with a set of requests.

Please note that your manuscript will not be scheduled for publication until you have made the required changes, so a swift response is appreciated.

IMPORTANT: The editorial review process is now complete. PLOS will only permit corrections to spelling, formatting or significant scientific errors from this point onwards. Requests for major changes, or any which affect the scientific understanding of your work, will cause delays to the publication date of your manuscript.

Should you, your institution's press office or the journal office choose to press release your paper, you will automatically be opted out of early publication. We ask that you notify us now if you or your institution is planning to press release the article. All press must be co-ordinated with PLOS.

Thank you again for supporting Open Access publishing; we are looking forward to publishing your work in PLOS Computational Biology. 

Best regards,

Boris S. Gutkin

Associate Editor

PLOS Computational Biology

Kim Blackwell

Deputy Editor

PLOS Computational Biology

***********************************************************

# Editor report: Acceptance letter

**DOI:** 10.1371/journal.pcbi.1008198.r004

**Authors:** Boris S. Gutkin, Kim T. Blackwell

10 Sep 2020

PCOMPBIOL-D-20-00531R1 

A comparison of neuronal population dynamics measured with calcium imaging and electrophysiology

Dear Dr Druckmann,

I am pleased to inform you that your manuscript has been formally accepted for publication in PLOS Computational Biology. Your manuscript is now with our production department and you will be notified of the publication date in due course.

The corresponding author will soon be receiving a typeset proof for review, to ensure errors have not been introduced during production. Please review the PDF proof of your manuscript carefully, as this is the last chance to correct any errors. Please note that major changes, or those which affect the scientific understanding of the work, will likely cause delays to the publication date of your manuscript. 

Soon after your final files are uploaded, unless you have opted out, the early version of your manuscript will be published online. The date of the early version will be your article's publication date. The final article will be published to the same URL, and all versions of the paper will be accessible to readers.

Thank you again for supporting PLOS Computational Biology and open-access publishing. We are looking forward to publishing your work! 

With kind regards,

Laura Mallard

PLOS Computational Biology | Carlyle House, Carlyle Road, Cambridge CB4 3DN | United Kingdom ploscompbiol@plos.org | Phone +44 (0) 1223-442824 | ploscompbiol.org | @PLOSCompBiol
